# Editorial: Endocrine complications of COVID-19: short and long

**DOI:** 10.3389/fendo.2025.1668201

**Published:** 2025-08-04

**Authors:** Kamyar Asadipooya

**Affiliations:** Division of Endocrinology, Diabetes, and Metabolism, Barnstable Brown Diabetes and Obesity Center, University of Kentucky, Lexington, KY, United States

**Keywords:** ACE2, complication, COVID-19, endocrine, management

The severe acute respiratory syndrome coronavirus 2 (SARS-CoV-2) infection mainly affects the respiratory system by attaching to its primary receptor ACE2 (angiotensin-converting enzyme 2), and using co-factors TMPRSS2 (the host proteases transmembrane protease, serine 2) and ADAM17 (A Disintegrin and Metalloproteinase 17) to gain cell entry. It can likewise invade other organs that carry ACE2 and these co-factors. Therefore, direct invasion is a recognized mechanism by which SARS-CoV-2 damages human tissues, including the endocrine structures. Furthermore, heightened inflammatory responses and cytokine production following acute SARS-CoV-2 infection could be responsible for multiple organ injuries and potential endocrine system dysfunction (1). However, the immune response following exposure to SARS-CoV-2 antigens are not always associated with endocrine organ dysfunction. For instance, immune response following vaccination was not significantly associated with endocrine system damage, including human reproductive system (Bao et al.). Moreover, post-COVID-19 syndrome (long COVID) is a debilitating problem after recovery from COVID-19, which can cause additional organ impairments and encompass adverse outcomes including disruption of endocrine-organ function (Zhang et al.). Consequently, it is necessary to recognize the risk factors, improve diagnostic tools and identifying more effective medications to prevent the short- and long-term complications of acute SARS-CoV-2 infection.

Increasing awareness among healthcare providers with regards to endocrine complications of acute SARS-CoV-2 infection is important. This can be achieved through reviewing current scientific literature and providing more evidence about diagnosis and treatment of COVID-19. The Research Topic “Endocrine Complications of COVID-19: Short and Long” contains four review and eight original research articles that discuss the important aspects related to endocrine organs and acute SARS-CoV-2 infection. The review articles summarize the connection between acute SARS-CoV-2 infection and thyroid dysfunction (Panesar et al.), diabetes development (Zhou et al.), adrenal damage and pituitary disruption (Iosef et al.) and long COVID in polycystic ovary syndrome (PCOS) patients (Zhang et al.). The research articles discuss the risk of hyponatremia based on CT findings in COVID patients (Wu et al.), and risk factors for long COVID in patients with type 2 diabetes (Matviichuk et al.). The connection between thyroid dysfunction and severe COVID-19 prognosis (Yang et al.), COVID severity (Zhang et al.), and long COVID (Dong et al.) are discussed separately.

Generally, management of acute SARS-CoV-2 infection is not limited to controlling viral replication and inflammation. We must consider the possible complications during acute infection or after recovery. The current therapeutic recommendation is mainly antivirals. However, this approach is probably facing a barrier, which is caused by the mutated forms of SARS-CoV-2. These mutations ultimately increase the transmission rate, escape the immune response following vaccination and endorse resistance to antiviral medications. Targeting ACE2 or other SARS-CoV-2 receptors may be a helpful strategy in reducing virus entry into host cells and mitigating severity of illness. Blocking virus interaction with its receptors or tackling receptors will bypass this defense mechanisms of SARS-CoV-2 mutated forms. This could be achieved by (1) reducing the interaction between ACE2 on cell membrane and SARS-CoV-2 spike protein (2), reducing the amount of soluble ACE2 or dipeptidyl peptidase 4 (DPP-4), which leads to less viral engulfment (3), capturing SARS-CoV-2 with a decoy receptor before entering into the cells, and (4) manipulating the expression or function of ACE2 genetically or with medications. However, ACE2 on cell membrane has protective roles and the fourth approach could be potentially harmful (2, 3).

In summary, providing more evidence about the complications and treatments of COVID-19 is necessary. Based on the possible complications, including long COVID, endocrine disruption etc., managing COVID-19 is not restricted to the treatment of acute SARS-CoV-2 infection and healthcare providers need to investigate the complications appropriately.
